# Exclusive Breastfeeding and Associated Factors among Mothers with Twins in the Tamale Metropolis

**DOI:** 10.1155/2020/5605437

**Published:** 2020-01-22

**Authors:** Rafatu Tahiru, Faith Agbozo, Hmphrey Garti, Abdulai Abubakari

**Affiliations:** ^1^Department of Nutritional Sciences, School of Allied Health Sciences, University for Development Studies, P. O. Box 1883, Tamale, Ghana; ^2^Yendi Nursing and Midwifery Training College, Ghana Health Service, Yendi, Ghana; ^3^University of Health and Allied Sciences, Ho, Ghana; ^4^Department of Public Health, School of Allied Health Sciences, University for Development Studies, P. O. Box 1883, Tamale, Ghana

## Abstract

**Background:**

Exclusive breastfeeding (EBF) for the first six months after birth has been recommended by the WHO as the best infant feeding strategy. Data on EBF rates among twin infants in Ghana remain limited and for that matter hypothesized to be low.

**Aim:**

The study sought to measure the prevalence of EBF and identify associated factors among twins in the Tamale metropolis.

**Methods:**

A cross-sectional survey involving 185 mother-twin pairs was conducted in four health facilities in the Tamale metropolis providing Child welfare Clinic (CWC) services. Socio-demographics data on both mother and twin were taken. Biomedical (e.g. perceived onset of lactation, confidence of producing enough milk, parity, delivery place, delivery type, time of breastfeeding initiation) and bio cultural factors (e.g. family cooperation for current infant feeding, breastfeeding counselling) were also obtained. In-depth interviews were also conducted with a sub sample of mothers (30) who were purposively selected to generate qualitative data on breastfeeding and associated cultural factors in twins as this data was necessary to aid in the explanation of the quantitative results.

**Results:**

Only 17% of twin infants were exclusively breastfed for six months. Women who were not confident that they could produce enough breast milk were about 83% less likely to practice exclusive breast-feeding (EBF) compared to those who were confident that they could produce enough breast milk (AOR = 0.17; CI = 0.04, 0.73; *p*-value = 0.017). Moreover, mothers who had no access to radio were about 87% less likely to practice EBF (AOR = 0.13; CI = 0.02, 0.79; *p*-value = 0.017). Moreover, mothers who had no access to radio were about 87% less likely to practice EBF (AOR = 0.13; CI = 0.02, 0.79;

**Conclusions:**

The study shows that, ownership of radio, confidence of producing enough breast milk and admission of the children into NICU were identified as the most important factors affecting exclusive breastfeeding of twins. Beyond Educating, encouraging and assuring twin mothers of their abilities to produce enough breast milk to satisfy their children, healthcare professionals should pay more attention on providing appropriate information on breastfeeding to mothers and caregivers.

## 1. Background

Exclusive breastfeeding without supplementation according to the World Health Organization and American College of Obstetricians and Gynecologists [1] is recommended for the first 6 months of life. The Ghana Child Health policy on exclusive breastfeeding also stresses on the fact that infants should be breastfed from birth till they are 6 months old [2].

Breast milk is nutritionally balanced, digests easily, confers immunity and promotes healthy growth [3]. Breast milk alone provides all of the nutrients, including vitamins and minerals infant needs, meaning that no other liquid or food is needed [4]. However infants living in Northern hemisphere may require vitamin D supplementation [5]. There is more evidence to suggest that if an infant is not breastfed it could lead to increased risks of preterm deaths, ill health in infants and young children [6]. To prevent 13–15% of 9 million deaths of children under-five in middle and low-income countries annually, a 90% universal coverage target for EBF is recommended by the WHO [7].

All children in Ghana (98%) are virtually breastfed at some point in their life. 2014 report of the GDHS [8] indicated that 52% of children younger than 6 months were exclusively breastfed. This shows an increase from the 46.3% (which represented a significant drop from the country's earlier achievement of 68% of exclusive breastfeeding coverage in 2012) recorded a few years ago, [9]. Odei [10] reported a 44% of exclusively breastfeeding rate for six months among singleton infants compared to 14% of twins who were EBF for six months in Accra. Other studies have also reported similar trends with twins and other higher order multiple births less likely to be exclusively breastfed for six months compared to their singleton counterparts [11, 12].

Another research which sought to evaluate the effectiveness of a Polish breastfeeding programme in the quest to promote EBF for six months among infants concluded that: EBF among twins and triplets was very low, 4.9% while that of the singletons was 73.2% [11].

Many unfavourable situations and issues concerning exclusively breastfeeding twins identified in some studies included: (a) repeated link of prematurity with multiple pregnancies, (b) lack/weakness of sucking reflex, (c) neurodevelopmental failure, and (d) separated due to stay in the intensive care [13]. In general socio-demographic and economic factors such as maternal age, education, employment and household income as well as infant feeding counselling and maternal and childcare services are also found to be important determinants of exclusive breastfeeding [14, 15].

Considering the importance of EBF to the health of both mothers and infants coupled with the relatively high twin birth rate in Ghana [16], it is crucial that mothers of twins are encouraged to exclusively breastfeed their infants to ensure optimal health. However, this cannot be done effectively without in-depth understanding of the factors that influence EBF among twins. Additionally, lack of information regarding breastfeeding practices among mothers of twins in Ghana makes it difficult to address any difficulties faced by such mothers. Hence the present study was designed to assess the prevalence of exclusive breast-feeding among twins and associated factors.

## 2. Methods

### 2.1. Study Area

This study was conducted at the Tamale Teaching hospital, Tamale West Hospital, Seventh Day Adventist Hospital and Central Reproductive and Child Health Center in the Tamale metropolis. Tamale Metropolis is the Regional capital of the Northern Region of Ghana. The Metropolis is boarded to the South by Central and East Gonja Districts, North by the Savelugu-Nanton District, East by the Mion District and West by Sagnerigu District. The 2012 and 2013 projected population of the Metropolis was 383205 and 404609 respectively [17].

### 2.2. Study Design, Population and Sampling

The study design was a mixed method, convergent design where both quantitative and qualitative data were collected simultaneously. The study was a facility based cross-sectional survey, which was conducted among mothers with twins 6–23 months in the Tamale Metropolis. A required sample size of 185 mothers of twins was calculated from 14% [18] prevalence of exclusive breastfeeding among twins in Greater Accra Region of Ghana using a 95% confidence interval and 5% margin of error. One hundred and eighty five mothers of twins between the ages of 6–23 months were selected for this study. They were drawn from the six Health facilities in the metropolis providing Child welfare Clinic (CWC) services on predetermined day(s) of the week. It was during these CWC services that the mothers of children age 6–23 months were selected using consecutive sampling, a non-probability sampling technique, as it was difficult to obtain large numbers of twins at a session, therefore in every visit we made to a selected facility on every child welfare session day, all mothers with twins who met the selection criteria were approached and when agreed to be part of the survey, were interviewed. This procedure continued in all selected facilities until the required sample size was achieved. This is because the mothers with twins at every child welfare session was small because twin delivery is not a very common occurrence hence random sampling was inappropriate. More so, the six (6) health facilities in each of the six sub-districts were selected on the basis of those that have very high numbers of mothers attending CWCs.

The 185 mother and child pairs were distributed among all the hospitals as a proportion of their respective CWCs daily attendance. Mothers with twins aged 6–23 months were interviewed in the present study. Mothers who were not breastfeeding at all due to personal choice or medical condition that interferes with breastfeeding such as mastitis were excluded as well as babies with any condition that made breastfeeding difficult (e.g. cleft palate).

The qualitative data was also generated from a sub sample of 30 mothers with twins who were purposively (based on educational status, ethnicity, etc.) selected to participate in the in-depth interviews. The sample aimed at a diversity of the participants' background (heterogeneous sampling). Five (5) mothers were selected from each of the six health facilities for this study.

### 2.3. Data Collection Methods

Mothers were interviewed using a semi-structured questionnaire. Data on maternal socio-demographics (e.g. age, marital status, occupation, income and highest educational level attained), biomedical (e.g. perceived onset of lactation, confidence of producing enough milk, parity, delivery place, delivery type, time of breastfeeding initiation) as well as health service and cultural factors (e.g. family cooperation for current infant feeding, breastfeeding counselling) were obtained.

Additionally, data on the infants' background characteristics (e.g. age in completed months, gestational age, Neonatal Intensive Care Unit admission and birth weight) were collected.

The interviews were conducted using the most dominant local language (Dagbani) in the study area on breastfeeding messages given by nurses after delivery, perception of inadequate milk production to satisfy their children for the first 6 months of life and cultural factors associated with breastfeeding in twins as this data was necessary to aid in the explanation of the quantitative results. The interviews were tape recorded. Before the interviews were conducted a research assistant explained the nature of the study to each woman in a separate room at the facility, read the consent form and obtained her verbal consent. Additionally, notes were taken for backup purposes and in order to ensure completeness of records.

### 2.4. Data Analysis

SPSS version 22.0 software (SPSS Inc. Chicago, IL, USA) was used for data entry and analysis. Responses on whether or not the children were given any feed aside breast milk in the first 6 months of life and the age (in months) at which water or other liquids was given were used to measure the prevalence of exclusive breastfeeding. Marital status, educational level, employment status, delivery type, decision to exclusively breastfeed, perceived onset of lactation, initiation of breastfeeding, confident of producing enough breast milk and breastfeeding counselling were crosstab with the prevalence of exclusive breastfeeding.

Descriptive statistics (frequencies and proportions) were used to summarize the demographic characteristics of participants. Chi-square was used to establish whether or not there were significant relationships between exclusive breastfeeding and maternal and infant factors. Logistic regression model was also estimated to determine predictors of exclusive breastfeeding among twins. We examined effect sizes of exposure variables as well as *p*-values of bivariate analysis before including the variables in the multivariate logistic regression model. Given the size of our study, variables that had *p*-values of 0.3 showed that the most effective and improved multivariable models were included. Even though marital status met the *p* ≤ 0.3 criteria, we did not include it in the multivariable modelling because they had poor distribution with exclusive breastfeeding and did not improve the models. For example, none of the mothers who were cohabiting practiced exclusive breastfeeding.

The qualitative data was analyzed using computer assisted qualitative data analysis software NVivo (version 10) (QSR international Pty Ltd, Doncaster, Victoria, Australia).

The field notes and audio recordings were translated verbatim. A thematic analysis was performed. The analysis started by reading, coding, and then categorizing the qualitative transcripts. Coding nodes were generated based on the study objectives and the main themes of the interview guide. Specifically, the coding was done by finding references to different ideas, concepts or categories in the form of sentences, phrases and paragraphs within the sources (transcript), which represented them. When a meaningful segment of the text was found, a code was assigned or category named to signify that particular segment. This continued until all the text was segmented. The process was repeated several times to make sure that all the relevant segments important to the study objectives were identified and coded.

Queries were used to find document content coded by a specific combination of nodes, or combination of nodes and attributes. They were made purposefully to identify content in the transcript with particular text so that they could be used as a basis for further analysis.

## 3. Results

### 3.1. Demographic and Socioeconomic Characteristics of Participants

The present study was conducted between April and July 2018.  Table 1 shows the demographic and socio-economic characteristics of the mothers. The mean age of the mothers was 30.18 ± 1.29/years. Majority of the mothers were married (83%), employed (85%) and lived in the urban areas (62%) of Tamale. About 66% had low level of education (primary and Junior High School education). About 51% had an average monthly income of less than GH₵200.00. Also 67% had Radio, 72% had television 67% had livestock while's 16% had a car. Most of the mothers lived in their own matrimonial homes.

### 3.2. Breastfeeding Information of Mother-Children Pair

Majority of twin infants (83%) were not exclusively breastfed for six months. Most of the mothers delivered in a health facility (85%), went through Caesarean section (62%) and were multiparous (51%). Majority of mothers (60%) were not confident they could produce enough milk to satisfy their children for the first six months of life. Breastfeeding initiation and perceived onset of lactation was 47% within 1 hour to 24 hours of birth and after 2 days of birth was 42%. Additionally, the decision to exclusively breastfeed prior to delivery was high (57%). Mothers who breastfed their babies between 8–12 times daily were 55% and the most popular reason (51.4%) for this behaviour was that breast milk was the only food they were surviving on. Majority (60%) of the mothers were not confident in producing enough breast milk to satisfy the children in the first 6 months of life (60%) and for that matter majority (83%) of the mothers did not practice exclusive breastfeeding (Table 2).

### 3.3. Twin Factors Associated with Exclusive Breastfeeding

Infants' sex, birth weight, gestational age and Suckling ability at birth were not significantly associated with EBF at six months. However, admission of infants at the neonatal intensive care unit (NICU) was significantly associated with exclusive breastfeeding at six months (*p* < 0.036) (Table 3).

### 3.4. Maternal Factors Associated with Exclusive Breastfeeding

Most mothers (61.4%) who did not practice exclusive breastfeeding for six months perceived that they could not produce enough breast milk to satisfy their infants until they were six months old.

Most of the maternal factors tested did not show any significant relationship with exclusive breastfeeding at six months (Table 4) except for ownership of radio and confidence to produce breast milk, which were significantly associated with exclusive breastfeeding (*p* = 0.041).

Women who were not confident that they could produce enough breast milk were about 83% less likely to practice exclusive breast-feeding (EBF) compared to those who were confident that they could produce enough breast milk (AOR = 0.1720; 95% CI = 0.04–0.79; *p*-value = 0.017). Moreover mothers who had no access to radio were about 87% less likely to practice EBF (AOR = 0.13; CI = 0.02–0.87; *p*-value = 0.027) (Table 5).

In the qualitative analysis three major themes were identified. They included reasons for a chosen daily frequency of breastfeeding, cultural factors and exclusive breastfeeding in twins and breastfeeding messages given during health education sessions.

### 3.5. Reasons for a Chosen Daily Frequency of Exclusive Breastfeeding

It was observed during the in-dept interviews with mothers that mothers who were breastfeeding their babies a certain number of times a day were doing so because they thought breastmilk was the only food for the baby. Except from the qualitative transcript bellow support this observation.

*“breast milk is the only food they depend on so I have to let them suckle many times besides I want them to grow fat”.*

However, others were of the view that the nature of their work limited their ability to breastfeed as frequently as possible as evidence in the excerpt below:

*“my work is so involving and I need to make money hence my inability to let them suckle much”, “I have other children to take care of so would not get enough time to let them suckle as frequently as possible”.*

### 3.6. Cultural Factors and Exclusive Breastfeeding

It was also observed during the in-depth interviews that cultural factors that affect breastfeeding have spiritual underpinnings. Evidence of this observation could be found in the except below:

*“there are spiritual connotations when one gives birth to twins hence needs to consult “elders” so you can care for them properly*. *At the time my children were eight (8) months old whiles one was walking properly the other was still crawling with no sign of walking anytime soon. I was advised to consult the “elders” which I did and I was told the children “wanted to go out”. Thankfully can't you see? He has also started to walk small small”. (32 years old mother at Tamale Central Reproductive and Child Health Center).*

Another mother also said* “one was always sending us back to the hospital, falling sick all the time whiles the other was fine. I was advised to see the “elders” which I did and was told one was “sitting on the other” so I needed to send them out to beg so he releases his sister. That is almost three (3) months now we haven't been to the hospital”. (28 years old mother at Tamale west Hospital).*

### 3.7. Messages Given during Breastfeeding Promotion

According to the mothers, health professionals gave them various health message on why they should practice exclusive breastfeeding despite the fact that they have twins. For most parts of the interview, mothers had similar health messages during child welfare sessions. These messages focus on why mothers should exclusively breastfeed their children. For instance, for the question “what were you told during breastfeeding promotion sessions?” A mother replied, “*The nurse said I should drink lots of fluid”*. Another mother also said *“the nurse said it was the best and most hygienic food for my children”*. Whiles several mothers' responses were in line with that of these two mothers, others had the following; *“I was told to eat more” and another said, “they told me to seek help from my relatives”.*

## 4. Discussions

The initial six months of life are critical for infant's health and development. Breast milk is the most useful source of nutrients that can be utilized during this period. Breastfeeding is more significant in multiple pregnancies, particularly since pre-term and low birth weight babies are frequently seen [19]. As a result, the Ghana Child Health Policy sought to promote exclusive breastfeeding from birth to six months and one of the strategies adopted to realise exclusive breastfeeding was the baby friendly hospital initiatives.

### 4.1. Prevalence of Exclusive Breastfeeding of Twins

In this study the prevalence and other associated factors of exclusive breastfeeding among mothers with twin babies were evaluated.

This study found an exclusively breastfeeding rate of 17% among mothers with twins in the Tamale metropolis. This observation is however higher than the 14% reported in Greater Accra Region by Jane (2013) [10] and 4.9% in Japan [11]. Difference in prevalence in different study populations could depend on different characteristics in its population or difference in time of study as national exclusive breastfeeding rate has increased marginally in recent times [7], a corresponding increase in EB in twins is likely.

The study investigated again the associated factors of EBF of twins and found two factors, which were statistically significantly associated with exclusive breastfeeding. They included: ownership of radio, and the confidence to produce enough breast milk to satisfy the children for the first six months of life.

Admission into NICU leads to delayed maternal attachment [20]. Findings also revealed that parents with an infant in the NICU experience depression, anxiety, stress, and loss of control, and they vacillate between feelings of inclusion and exclusion related to the provision of health care to their neonate [21]. Lactation insufficiency frequently is blamed on stresses, such as those imposed by preterm delivery, infant medical condition, or maternal lifestyle [22]. Mothers tend to look for alternatives to feed their children. This could explain why majority (58.9%) of the mothers who had either one or both of their children admitted into the Neonatal Intensive Care Unit did not practice exclusive breastfeeding. This observation is in accordance with findings of Weimers and colleagues [3] who reported that, 70% of mothers who did not practice exclusive breastfeeding as a result of NICU admission. In contrast Maastrup and friends [23] research in Denmark found 68% of mothers practicing exclusive breastfeeding at NICU. Perhaps the difference could be as a result of the knowledge level and commitment of both mothers and health staff at NICU. Also some mothers could have had a bed in the NICU when she was discharged from maternity ward making it easier for rooming-in.

Mass media could help by putting the issue of breastfeeding on policy agendas and by framing breastfeeding as healthy and normative for baby and mother.

Radio is an easy and effective means of mass media. In many developing countries, there is evidence that health education and information via radio broadcasting can improve public health. For example, in Ghana the current national child nutrition campaign on TV and radio is the United States Agency for International Development (USAID) sponsored advert “Aduane Pa Ma Asetena Pa” (Good Food for Good Life). The advert which was launched in 2013 by the Ministry of Health and the Ghana Health Service airs on Ghanaian TV and radio channels and mainly focuses on breastfeeding and appropriate and timely complementary feeding. The radio programme is an integral component of the integrated 1,000-day household approach in Ghana [23]. Thus, it is possible that information from this programme might have influenced mothers to practice EBF [24] as mothers who had access to radio were more likely to practice EBF. A greater proportion of mothers who owned a radio set (58.8%) in this study exclusively breastfed their children. Similar findings have been observed in a research by Foss & Southwell, 2006 [25] who concluded that Mass media content likely influences the decision of women to breastfeed their new-born children. Moreover, Tamiru et al. [26] found that in rural Ethiopia women who own radio were more likely to practice exclusive breastfeeding compare to those who do not have. Similarly, in Morogoro Tanzania another study by Shimiri et al. [27] showed that possession of radio was positively associated with exclusive breastfeeding. The ownership of radio in this context is not a proxy for wealth but as a source of information, as mass media channels are often used to communicate information on health and nutrition in Ghana.

Contrary to this study, Jane's [9] research in the Greater Accra region found no significant relationship (*p* value = 0.106) between owning a radio and exclusive breastfeeding. This could be as a result of the Geographical location. Where there are many radio stations to tune into, it becomes difficult to get people's attention on an educative health Programme.

Majority (61%) of mothers in this study who were not confident of producing adequate breast milk to satisfy their infants till they turned six months introduced other foods and liquids particularly formula and porridge before six months. Jane's study [9] corroborated the findings of the present study. For instance she showed that 90% of mothers were not confident of exclusively breastfeeding their children for 6 months introduced other foods before 6 months. Similar observations was made by Lela M. Chartman and friends [3] in 2004 where they suggested that, the dominant reason for partial breast feeding (76%) was as a result of maternal anxiety that breast milk alone might not provide sufficient nourishment for the children.

The perception of breast milk insufficiency has been reported by other researchers to be a major barrier to EBF even among singletons [28–30]. More so a study conducted in Alberta and Calgary revealed that high milk production among mothers was associated with perceived confidence of mothers in breastfeeding [31]. This observation is in line with our finding which show that Perceived confidence of mothers to produce enough milk for their twin babies was positively associated with exclusive breastfeeding. This finding underscores the importance of mother's emotional and psychological stability in ensuring adequate milk production, which is crucial in ensuring successful exclusive breastfeeding of infants in the first six months of life. More so, scientific researches demonstrate that the production of milk in case of mothers of twins is doubled. Six months after childbirth a body of a mother of twins can produce from 1.0 to 2.0 kg of milk per day, while a body of a mother of triplets, more than 3.0 kg [32].

To do this however, they should trust in themselves, get enough rest, ensure they are well fed, get support from people close to them, and try to ensure that their baby's suction power is sufficient.

In this study, 55% of mothers breastfed their infants 8 to 12 times daily. This is consistent with the recommendation [33] that breastfeeding frequency impacts the initiation of lacto genesis II, which influences duration of exclusive breastfeeding and concluded that breastfeeding should be done as frequent as eight to twelve times a day. In Ghana, the breastfeeding policy advocates that mothers breastfeed at least eight to twelve times per 24 h period to ensure that babies receive enough milk [2].

In addition to this, majority of the mothers (85%) in the present study engaged in some form of employment so are unable to exclusively breastfeed because facilities at their work places and conditions of work do not support exclusive breastfeeding. Even though maternal work and exclusive breastfeeding does not seem well-matched, not all-maternal work are incompatible with the practice of exclusive breastfeeding [34]. Some of the reasons accounting for the frequency of breastfeeding daily included: *“that was the only food they survive on”, “I have to work for money”, “I have other children to care for”, “I want them to grow fat” and they naturally eat a lot”.*

Further analysis of the data also showed the existence of belief systems and practices (twins), which had a connection to exclusive breastfeeding. Most of those we interacted with attested positively to the fact that, their belief in the advice of the “elders” (beg for alms) really worked for them. Not all the respondents reported observed the practice in their families. Those who did not observe such practice had either abandoned the traditional account of disease aetiology or were motivated by their faith (Islam or Christianity). Being on the streets exposes them to all sorts of readily available foods and drinks, some passers-by also give the children other gifts, which could include food or drinks hence the higher the possibility of not practicing exclusive breastfeeding.

Also, according to the mothers, health professionals gave them various health messages on why they should practice exclusive breastfeeding despite the fact that they have twins. For most parts of the interview, mothers had similar health messages during child welfare sessions. This observation was in line with the initiative adapted in Ghana to promote exclusive breastfeeding-“the Baby Friendly Hospital Initiative”. All the facilities selected for the present study were designated baby friendly and were expected to help mothers initiate breastfeeding within one and half-hour of birth, guide the mothers on how to breastfeed and maintain lactation, even if they should be separated from their infants and also encourage mothers to give newborn infants no food or drink other than breastmilk, unless medically indicated. They were also expected to inform all pregnant women about the benefits and management of breastfeeding. Our observations confirmed that the selected facilities were up to their task and could have a profound influence on the decision of mothers to practice exclusive breastfeeding and ensure continuation of breastfeeding. This was meant to prevent mothers from early introduction of other foods and liquids, which could increase the family's cost of caring for the babies taking into consideration the fact that most of the mothers earn less than GH₵200.00 (51%). This could also result in diarrhoea, otitis media and other infections [35].

Findings from the present study should be interpreted with caution as retrospective crossectional studies has inherent weakness such as recall bias, which was inevitable but all efforts were made to make sure the effect of the recall bias was minimal. The study sample may not also give us power to model more variables and detect meaningful effects. However, as studies exploring these outcomes are not common in the area, these findings could be useful in generating hypotheses, which could be explored in larger future studies.

## 5. Conclusion

On the whole, the study revealed a 17% Exclusive breastfeeding rate among mothers with twins in the Tamale metropolis. The practice appeared to be largely dependent on Admission of infants into the Neonatal Intensive Care Unit (NICU), mothers' confidence to exclusively breastfeed and the possession of radio. Those who received support in the form of approximate information breastfeeding were also more likely to practice EBF than those who did not. Cultural factors played a huge role in the practice of EBF and raising of twins.

Breastfeeding of multiples despite the many potential advantages is a difficult and stressful challenge, which should be approached with, appropriate sensitivity and understanding as observed in this study.

Beyond Educating and Encouraging twin mothers of their abilities to produce enough breast milk to satisfy their children, healthcare professionals should pay more attention to providing mothers and caregivers with appropriate information on breastfeeding.

## Figures and Tables

**Table 1:** Demographic and socioeconomic characteristics of mothers (*N* = 185).

Variable	Frequency	Percentage (%)
*Mother's age (in years)*		
<20 years	50	27.0
21–35 years	105	57.0
>35years1	30	16.0

*Marital status*		
Single	11	6.0
Married	154	83.0
Co-habiting	20	11.0

*Educational level*		
Low educational level	122	66.0
High educational level	63	34.0

*Employment status*		
Employed	157	85.0
Unemployed	28	15.0
*Residence*		
Urban	115	62.0
Rural	70	38.0

*Average monthly income*		
<GH₵200	95	51.0
≥GH₵200	44	24.0
Don't know	46	25.0

*Ownership of a house*		
Own house	87	47.0
Rented apartment	43	23.0
Family house	56	30.0

*Ownership of a Car*		
Yes	30	16.0
No	155	84.0

*Ownership of television*		
Yes	30	72.0
No	120	28.0

*Ownership of livestock*		
Yes	124	67.0
No	61	33.0

*Ownership of radio*		
Yes	124	67.0
No	54	33.0

**Table 2:** Breastfeeding information of mother-children pair (*N* = 185).

Variable	Frequency	(%)
*Place of delivery*		
Facility based	157	85.0
Home	28	15.0

*Delivery type*		
Vaginal delivery	70	38.0
Caesarean section	115	62.0

*Parity*		
Primaparous	91	49.0
Multiparous	94	51.0

*Initiation of breastfeeding*		
<30 mins	6	3.0
30–1 hr	39	21.0
1–24 hrs	78	42.0
>1 day	63	34.0

*Perceived onset of lactation*		
1 day	28	15.0
2 days	87	47.0
3 days	59	32.0
>3 days	7	6.0

*Breastfeeding counselling*		
Yes	85	46.0
No	100	54.0

*Decision to EBF after delivery*		
Yes	105	57.0
No	80	43.0

*Frequency of EBF daily*		
<8times	26	14.0
8–12times	102	55.0
>12times	57	31.0

*Confident of producing enough milk*		
Yes	74	40.0
No	111	60.0

*Practice of exclusive breastfeeding*		
Yes	31	17.0
No	154	83.0

EXB: exclusive breastfeeding.

**Table 3:** Infants factors associated with exclusive breastfeeding.

Variable	Exclusive breast feeding (%)		*p*-value
	No (*N* = 154)	Yes (*N* = 31)	
*Gender of children*			
Male	45.5	52.6	0.376
Female	54.5	47.4	

*Gestational age*			
Pre term	55.4	47.1	0.714
At term	43.4	52.9	
Post term	1.2	0.0	

*Birth weight (1^st^ twin)*			
LBW	62.7	58.8	0.764
NBW	37.3	41.2	

*Birth weight (2^nd^ twin)*			
LBW	53.0	54.5	0.374
NBW	44.0	45.5	

*Admission into NICU*			
No	18.1	41.2	0.036
Yes	81.9	58.8	

*Suckling ability at birth*			
Poor	48.8	51.2	0.425
Normal	51.2	48.8	

*p*-value <0.05.

**Table 4:** Maternal factors associated with exclusive breastfeeding.

Variable	Exclusive breast feeding		*p*-value
	No (*N* = 154)	Yes (*N* = 31)	
*Mother's age (in years)*			
<20 years	10.0	0.0	0.301
21–35 years	42.0	9.0	
>35 years	31.0	8.0	

*Marital status*			
Single	6.0	5.9	0.278
Married	80.7	94.1	
Co-habiting	13.3	0.0	

*Educational level*			
Low educational level	65.1	70.6	0.661
High educational level	34.9	29.4	

*Employment status*			
Employed	88.0	70.6	0.068
Unemployed	12.0	29.4	

*Ownership of television*			
Yes	74.7	58.8	0.184
No	25.3	41.2	

*Ownership of radio*			
Yes	74.7	58.8	0.041
No	25.3	41.2	

*Initiation of breastfeeding*			
<30 mins	2.4	5.9	0.702
30–1 hr	20.5	23.5	
1–24 hrs	41.0	47.1	
>1 day	36.1	23.5	

*Perceived onset of lactation*			
1 day	12.0	29.4	0.245
2 days	47.0	47.1	
3 days	34.9	17.6	
>3 days	6.1	5.9	

*Delivery type*			
Vaginal delivery	36.1	47.1	0.398
Caesarean section	63.9	52.9	

*Breastfeeding counselling*			
No	54.2	52.9	0.923
Yes	45.8	47.1	

*Decision to EBF after delivery*			
No	39.8	58.8	0.148
Yes	60.2	41.2	

*Confident of producing enough milk*			
No	61.4	35.3	0.047
Yes	38.6	64.7	

*Average monthly income*			
<GH₵200	18.0	82	0.237
≥GH₵200	12.5	87.5	
Don't know	28.0	72.0	

**Table 5:** Determinants of exclusive breastfeeding at 6 months (*N* = 185).

Variable	Odds ratio	*p*-value	95% CI
*Ownership of radio*			
No	0.13	0.027	0.02–0.79
Yes	Reference		

*Decision to EBF after delivery*			
No	2.93	0.104	0.80–10.73
Yes	Reference		

*Employment status*			
Unemployed	1.29	0.737	0.29–5.67
Employed	Reference		

*Breastfeeding education*			
No	1.12	0.865	0.30–4.26
Yes	Reference		

*Confident of producing enough milk*			
No	0.17	0.017	0.04–0.73
Yes	Reference		

*Initiation of breastfeeding*			
<30 mins	3.64	0.418	0.16–82.96
30–1 hr	Reference		
1–24 hrs	1.87	0.451	0.37–9.51
>1 day	1.16	0.874	0.18–7.55

*Perceived onset of lactation*			
1 day	1.48	0.674	0.24–9.22
2 days	Reference		
3 days	0.25	0.151	0.06–1.56
>3 days	0.51	0.467	0.03–5.17

*Suckling ability at birth*			
Poor	0.67	0.565	0.17–2.63
Normal	Reference		

*Admission into NICU*			
No	0.29	0.088	0.07–1.20
Yes (Ref)			

No. of obs. = 185, LR Chi2 (11) = 17.98, Prob. > Chi2 = 0.0821, Pseudo R2 = 0.1972, Ref: Reference category; CI: Confidence Interval; ^∗^*p* < 0.05.

## Data Availability

The data supporting the conclusions of this article are included within the manuscript. Upon reasonable request, the dataset could be obtained from the corresponding author.

## Abbreviations

ANC: Antenatal care
BF: Breastfeeding
EXB: Exclusive breastfeeding
GDHS: Ghana demographic and health survey
IYCF: Infant and young child feeding
NICE: National institute of clinical excellence
NICU: Neonatal intensive care unit
UNICEF: United nations international children's emergency fund
USBC: United states bowling congress
WHO: World health organization.
